# The Therapeutic Effects of Exosomes Derived from Human Umbilical Cord Mesenchymal Stem Cells on Scleroderma

**DOI:** 10.1007/s13770-021-00405-5

**Published:** 2021-11-16

**Authors:** Yue Yu, Liangliang Shen, Xiaoyun Xie, Jingjun Zhao, Miao Jiang

**Affiliations:** 1 Department of Dermatology, Tongji Hospital, School of Medicine, Tongji University, Shanghai, 200065 China; 2 Department of Interventional and Vascular Surgery, Shanghai Tenth People’s Hospital, School of Medicine, Tongji University, Shanghai, 200072 China

**Keywords:** Scleroderma, Human umbilical cord mesenchymal stem cell, Exosome, Macrophage, Bleomycin

## Abstract

**Background:**

Scleroderma is a multisystem disease in which tissue fibrosis is caused by inflammation and vascular damage. The mortality of scleroderma has remained high due to a lack of effective treatments. However, exosomes derived from human umbilical cord mesenchymal stem cells (HUMSCs)-Ex have been regarded as potential treatments for various autoimmune diseases, and may also act as candidates for treating scleroderma.

**Methods:**

Mice with scleroderma received a single 50 μg HUMSCs-Ex. HUMSCs-Ex was characterized using transmission electron microscopy, nanoparticle tracking analysis and nanoflow cytometry. The therapeutic efficacy was assessed using histopathology, immunohistochemistry, immunofluorescence, quantitative real-time polymerase chain reaction, enzyme-linked immunosorbent assay and western blot.

**Results:**

HUMSCs-Ex ameliorated the deposition of extracellular matrix and suppressed the epithelial-mesenchymal transition process, and the effects lasted at least three weeks. In addition, HUMSCs-Ex promoted M1 macrophage polarization and inhibited M2 macrophage polarization, leading to the restoration of the balance of M1/M2 macrophages.

**Conclusion:**

We investigated the potential antifibrotic and anti-inflammatory effects of HUMSCs-Ex in a bleomycin-induced mouse model of scleroderma. So HUMSCs-Ex could be considered as a candidate therapy for scleroderma.

**Supplementary Information:**

The online version contains supplementary material available at 10.1007/s13770-021-00405-5.

## Introduction

Scleroderma is an autoimmune tissue disease characterized by vascular dysfunction, inflammatory disorder and extensive fibrosis [1]. Although the prevalence (7 ~ 489/1 million) and the incidence of (0.6 ~ 122/1 million) scleroderma are low [2], the death rate remains high [3]. The 10-years survival rate is only 66%, and the quality of life of survivors is significantly affected [4]. The main reason for the poor outcomes of scleroderma is that fibrosis destroys the normal structures of the skin, lung, kidney and skeletal system, resulting in severe complications such as pulmonary hypertension, acute kidney injury and acro-osteolysis [5].

Macrophages are involved in the process of scleroderma [6]. After infiltrating the tissue, monocytes can differentiate into macrophages depending on the microenvironment. Differentiated macrophages can be divided into classical activated inflammatory macrophages (M1) and tissue-activated fibrotic macrophages (M2). M1 macrophages promote inflammation by releasing TNF-α, IL-6, and IL-12. M2 macrophages stimulate fibrosis by releasing IL-4, IL-10 and IL-13. Overall, regulation of the M1/M2 macrophage balance is a strategy for the treatment of scleroderma [7]. Epithelial-mesenchymal transition (EMT) is a process in which stable epithelial cells lose cell–cell adhesion and acquire mesenchymal characteristics, and the main mesenchymal cells in skin are fibroblasts. Activation of fibroblasts is an important process of promoting extracellular matrix (ECM) deposition, which is realized by differentiation into myofibroblasts [8].

Stem cells are a kind of cell with the potential for self-renewal and multidirectional differentiation, so they have been used in the repair of various tissues and organs. Multiple studies have shown that mesenchymal stem cells (MSCs) mainly rely on paracrine factors to play a therapeutic role, and exosomes are an important component of paracrine factors [9, 10]. Exosomes are vesicles filled with a variety of proteins, microRNAs, and DNA that perform biological functions on target cells [11]. In addition, HUMSCs-Ex transplantation is a cell-free therapy, that has the characteristics of low immune source and high stability [12]. Studies have shown that exosomes can treat various diseases by regulating EMT [13] or macrophage balance [14], but the mechanism of exosomes in scleroderma remains unclear. In this study, we confirmed that HUMSCs-Ex had an effect on the treatment of skin fibrosis and the regulation of macrophage balance in a mouse model of scleroderma.

## Materials and methods

### Exosomes purification and characterization

Human umbilical cord mesenchymal stem cells were obtained from Shanghai Tenth People’s Hospital and cultured in DMEM/F12 complete medium (Thermo Fisher Scientific, Shanghai, China) at 5% CO_2_ and 37 °C. The cells at passage four were used for experiments. The cell culture supernatant was centrifuged at 2000 × g for 30 min to eliminate cell debris. After centrifugation at 10,000 × g for 45 min, the supernatant was filtered through a 0.22-μm filter (Millipore, Burlington, MA, USA). The supernatant was transferred to new tubes and centrifuged at 100,000 × g for 70 min in an ultracentrifuge (Hitachi, Tokyo, Japan). After a second ultracentrifugation, exosomes were resuspended in 100 μL of cold PBS and taken for subsequent identification. The concentration of HUMSCs-Ex was measured using a BCA protein assay kit (Beyotime, Shanghai, China). Transmission electron microscopy (TEM)(Hitachi), nanoparticle tracking analysis (NTA)(NanoFCM, Xiamen, China) and nanoflow cytometry (nFCM)(NanoFCM) were used to analyze the morphology and surface markers of HUMSCs-Ex.

### Preparation of a mouse model of scleroderma

Female Balb/c mice aged 6 to 8 weeks were purchased from Shanghai SLAC Laboratory Animal Center. Female Balb/c mice aged 6 to 8 weeks were purchased from Shanghai SLAC Laboratory Animal Center. We did not explore the effects of the gender of the mice on results, but female mice are commonly used in the bleomycin-induced mouse model of scleroderma. Mice were randomly divided into the control group and the bleomycin-induced group. Mice in the bleomycin-induced group were subcutaneously injected with 100 μl bleomycin (Thermo Fisher Scientific) at 1 mg/mg once a day for 4 weeks. Mice in the control group were injected with 100 μL phosphate buffered saline (PBS) for 4 weeks. On day 28, the bleomycin-induced group was randomly divided into the exosome (Ex) group and the new bleomycin-induced (BLM) group. Mice in the Ex group were subcutaneously injected on their backs with 100 μL of PBS containing 50 μg of HUMSCs-Ex, while the control group and BLM group were injected with 100 μL PBS. Mice were randomly sacrificed, and skin and blood samples were collected at 1, 2, and 3 weeks after HUMSCs-Ex or PBS injection.

### Histological assessment

Specimens from skin lesions were fixed overnight with 4% paraformaldehyde for 24 h at 4 °C, paraffin-embedded and sectioned at 4 μm. The sections were stained with hematoxylin & eosin (H&E) or Masson’s trichrome to assess the dermal thickness and collagen content, respectively. The histologic sections were scanned by pannoramic scan (3DHISTECH, Budapest, Hungary). Then the images were observed and photographed using CaseViewer software (3DHISTECH, Hungary) at 100 × magnification and quantified in Image J software (National Institutes of Health, Bethesda, MD, USA). Three microscopic fields were selected for each section. Dermal thickness was defined as the distance between the epidermal-dermal junction and the dermal-subcutaneous fat junction. Collagen content was defined as the percentage of collagen deposition area to total area.

### Immunohistochemical analysis

The paraffin-embedded skin sections were incubated with 3% H_2_O_2_ for 10 min and then 10% normal goat serum albumin (Solarbio, Beijing, China) for 10 min. The sections were stained with mouse monoclonal antibodies against α-SMA (1:200 dilution, BOSTER, Wuhan, China), Vimentin (1:200 dilution, BOSTER), E-cadherin (1:200 dilution, Cell Signaling Technology, Danvers, MA, USA) and TGF-β1 (1:200 dilution, BOSTER) overnight at 4 °C. The sections were incubated with biotinylated secondary antibodies for 30 min at room temperature. Diaminobenzidine solution was used as a chromogen to visualize the immunostaining results. The immunohistochemistry sections were scanned by pannoramic scan. Then, the images were observed and photographed using CaseViewer software at 400 × magnification, and quantified in Image-Pro Plus software (Media Cybernetics, Rockville, MD, USA). Five microscopic fields were selected for each section, and the mean intensity optical density (IOD) was used to quantify the protein expression level.

### Immunofluoresence analysis

The paraffin-embedded skin sections were blocked with 10% normal goat serum albumin and incubated with mouse anti-iNOS antibody (1:200 dilution, Abcam, Cambridge, MA, USA) or rabbit anti-CD206 antibody (1:200 dilution, Abcam), and rabbit anti-F4/80 antibody (1:200 dilution, Abcam) overnight at 4 °C. The sections were incubated with Alexa Fluor 488 AffiniPure goat anti-rabbit IgG (1:500 dilution, Jackson ImmunoResearch, West Grove, PA, USA) or Alexa Fluor 488-conjugated AffiniPure goat anti-mouse IgG (1:500 dilution, Jackson ImmunoResearch) and Cy3-conjugated AffiniPure goat anti-rabbit IgG (1:500 dilution, Jackson, USA) at for 1 h. Then nuclei were stained with 6-diamidino-2-phenylindole. The sections were observed by fluorescence microscopy (Nikon, Shanghai, China) and scanned by pannoramic scan. Then the double positive cells were counted by CaseViewer software at 630 × magnification and five microscopic fields were selected for each section.

### Cytokine analysis by enzyme-linked immunosorbent assay (ELISA)

The levels of IL-4, IL-6, IL-10 and IL-12p40 in serum were quantified by ELISA kits (BOSTER) according to the manufacturer’s instructions. The absorbance was determined at 450 nm by an ELISA reader (Rayto, Shenzhen, China) and the actual concentration was then calculated using the standard curve.

### Quantitative real-time polymerase chain reaction (qPCR)

Total RNA was extracted from skin samples using TRIzol reagent (Invitrogen, Waltham, MA, USA). Complementary DNAs (cDNAs) were reverse transcribed using the PrimeScript™ RT reagent Kit (TaKaRa, Japan). qPCR was performed with AceQ Universal SYBR qPCR Master Mix (Vazyme Biotech Co.,Ltd, Nanjing, China) in a BIOER 9600 real-time PCR system. Target gene expression levels were calculated using the 2 − ΔΔCt method, and were normalized to ACTB. The primers used were as follows: col1α1,  5′-CTGGCGGTTCAGGTCCAAT-3′ (forward primer) and 5′-TTCCAGGCAATCCACGAGC-3′ (reverse primer); col3α1, 5′-CTGTAACATGGAAACTGGGGAAA-3′ (forward primer) and 5′-CCATAGCTGAACTGAAAACCACC-3′ (reverse primer); ACTB, 5′-GTGACGTTGACATCCGTAAAGA-3′ (forward primer) and 5′-GCCGGACTCATCGTACTCC-3′ (reverse primer).

### Western blot

Skin samples were lysed with RIPA buffer (BOSTER) and PMSF (BOSTER) to extract protein. The concentration of protein was determined with a BCA protein assay kit (BOSTER). An equivalent amount of protein was loaded onto 10% SDS-PAGE gels (BOSTER), electrophoretically treated and then transferred to PVDF membranes (Millipore). The membranes were incubated with 5% nonfat milk in TBS-T for 2 h at room temperature, and then incubated with primary antibodies at 4 °C overnight. The membranes were incubated with secondary antibodies for 2 h at room temperature, and the protein bands were visualized using ECL reagents. Finally, the bands were photographed using gel image analysis system (Tanon Science & Technology Co., Shanghai, China) and the intensity of the bands was quantified in Gel-Pro analyzer software (Media Cybernetics). The details of the primary and secondary antibodies are as follows: anti-iNOS (1:1000, Thermo Fisher Scientific), anti-Arg-1 (1:500, BOSTER), and horseradish peroxidase-conjugated AffiniPure goat anti-rabbit IgG (1:2000, Jackson ImmunoResearch).

### Statistical analysis

All statistical analyses were performed using GraphPad Prism software version 8.0.1 (GraphPad Software, San Diego, CA, USA). One-way ANOVA was used to analyze the significant differences when multiple experimental groups were compared. *P* values < 0.05 were considered statistically significant. Data are presented as the mean ± SD.

## Results

### Characterization of HUMSCs-Ex

**Fig. 1:**
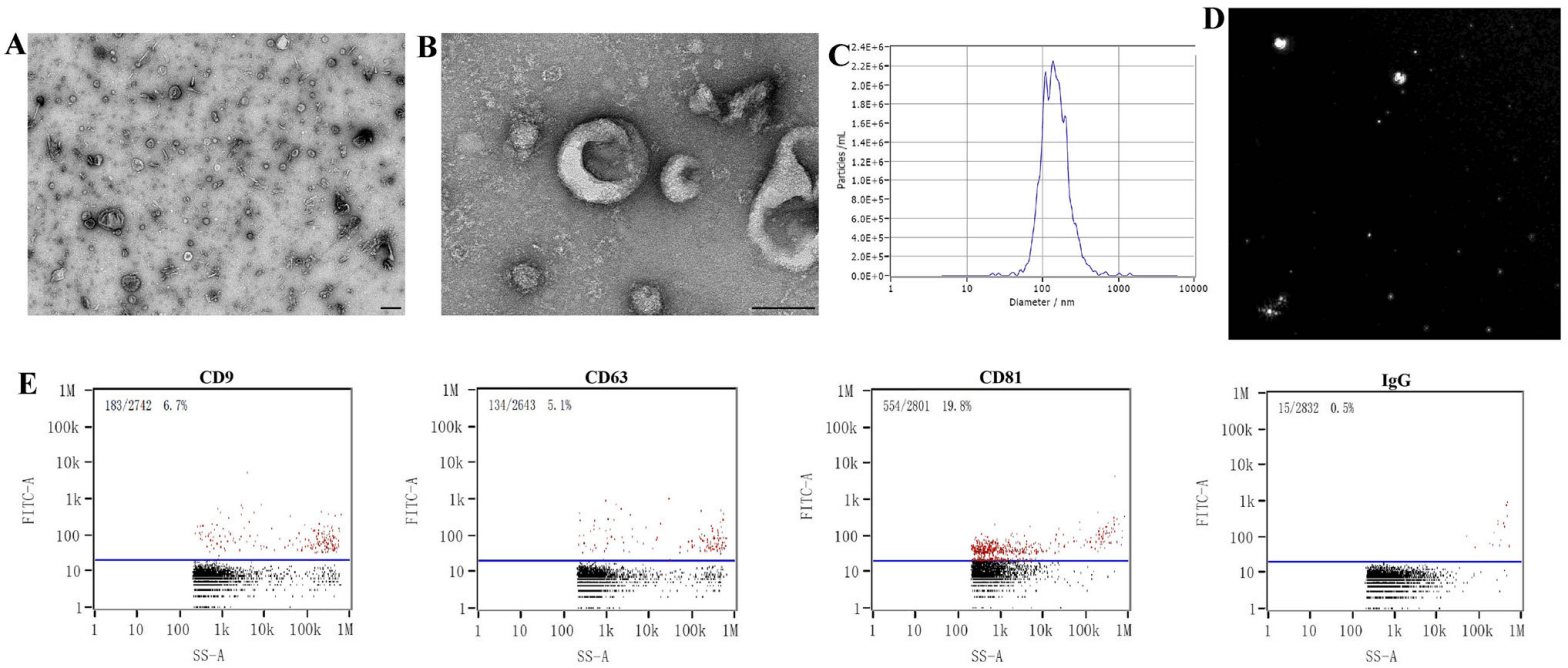
**A**, **B** Identification of HUMSCs-Ex. Representative images of HUMSCs-Ex under TEM. Scale bars = 200 nm (**A**) and 100 nm (**B**). **C** NTA of HUMSCs-Ex diameters. **D** NTA image of HUMSCs-Ex. **E** CD9, CD63 and CD81 expression on single HUMSCs-Ex using FITC by nFCM. An IgG isotype control was used to establish the nonspecific binding of antibodies

TEM images showed that HUMSCs-Ex presented a globulare shape in Fig. 1 and a saucer-shaped morphology, as shown in Fig. 1B. NTA showed that the mean diameter of HUMSCs-Ex was 140.6 nm (Fig. 1C), which was consistent with previously reported exosomes. nFCM revealed that the expression levels of CD9, CD63 and CD81 were obviously higher than that of the IgG isotype control (Fig. 1D).

### HUMSCs-Ex ameliorated skin fibrosis in a mouse model of scleroderma

**Fig. 2:**
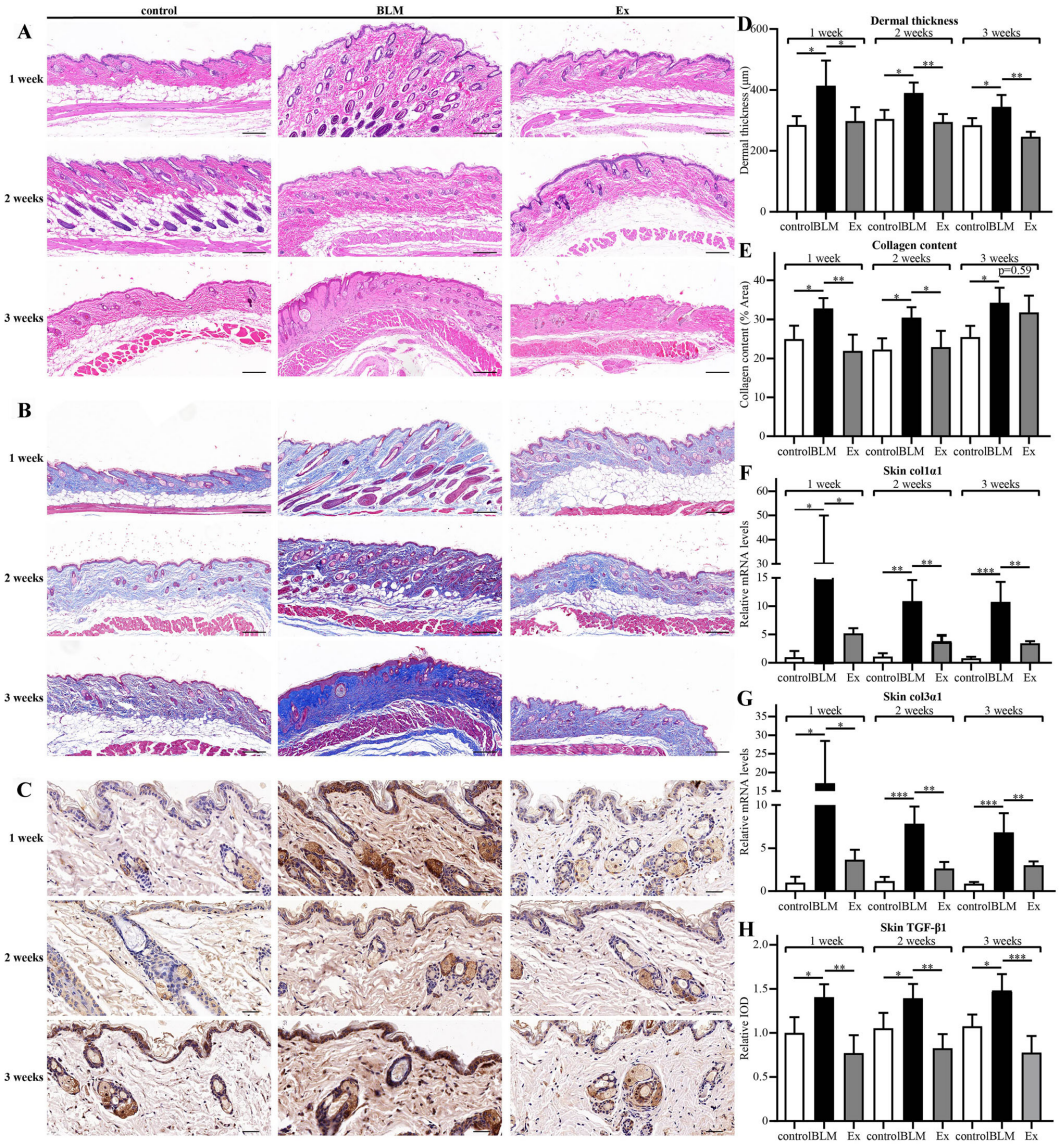
HUMSCs-Ex alleviated skin fibrosis in a bleomycin-induced mouse model of scleroderma. **A**, **B** The respective images are the skin sections stained with H&E and Masson’s trichrome. Scale bars = 200 μm. **C** The distribution and expression of TGF-b1 were assessed by immunohistochemical staining. Scale bars = 40 μm. **D**–**E** Dermal thickness and collagen content were quantified from H&E and Masson’s staining respectively. **F**–**G** Col1α1 and col3α1 related to ECM were assessed by qPCR. **H** The protein expression level was quantified with IOD. Data are presented as the mean ± SD, *p < 0.05, **p < 0.01, and ***p < 0.005

As shown in Fig. 2, the epidermis, dermis and subcutaneous fat layer were clearly differentiated in the control group, and there was no inflammatory cell infiltration. Compared with the control group, the dermal thickness increased significantly and the subcutaneous fat layer partially disappeared in the BLM group. HUMSCs-Ex reduced the dermal thickness (Fig. 2D). As shown in Fig. 2B, collagen fibers were arranged regularly in the control group. Compared with the control group, collagen fibers in the BLM group were thickened and replaced the subcutaneous fat layer to some extent. HUMSCs-Ex significantly reduced the collagen content at 1 week and 2 weeks but not at 3 weeks, which might be caused by HUMSCs-Ex not restoring the subcutaneous fat layer (Fig. 2E). Collagen is a marker of ECM and is used to assess the extent of collagen deposition. As shown in Fig. 2F–G, the relative expression of col1α1 and col3α1 mRNA in the BLM group were higher than that in the control group. HUMSCs-Ex reduced the transcription level of col1α1 and col3α1 mRNA. TGF-β1 is believed to be a central mediator in skin fibrosis and a biomarker for predicting the progression of scleroderma [19]. Compared with the control group, the IOD score of TGF-β1 was elevated in the BLM group (Fig. 2C). HUMSCs-Ex reduced the expression of TGF-β1 (Fig. 2H). The above changes in dermal thickness, and TGF-β1, col1α1 and col3α1 mRNA expression levels were similar except for collagen content at 1 week, 2 weeks and 3 weeks.

### HUMSCs-Ex inhibited skin EMT in a mouse model of scleroderma

**Fig. 3:**
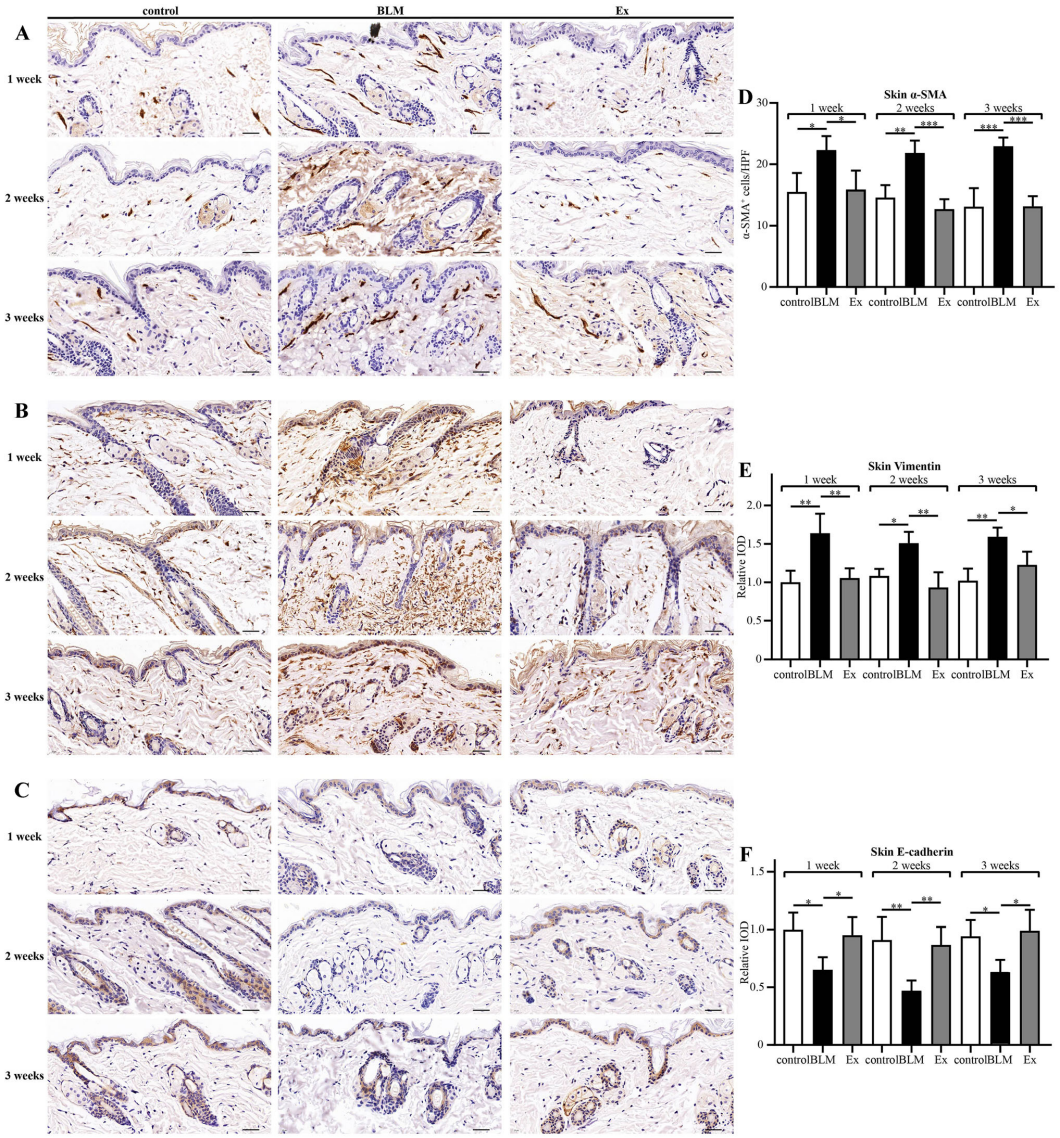
HUMSCs-Ex restored EMT. **A**–**C** The distribution and expression of α-SMA, vimentin and E-cadherin were assessed by immunohistochemical staining. Scale bars = 40 μm. **D** The number of α-SMA^+^ myofibroblasts was counted under each high power field. **E**–**F** The protein expression levels of vimentin and E-cadherin were quantified with IOD. Data are presented as the mean ± SD, *p < 0.05, **p < 0.01, and ***p < 0.005

To explore the influence of HUMSCs-Ex on EMT, we performed immunohistochemistry analysis of E-cadherin, a marker of endothelial tissue, and Vimentin, a marker of mesenchymal tissue. We also studied α-SMA, a marker of myofibroblasts. The immunohistochemistry results revealed an increased expression of Vimentin and a decreased expression of E-cadherin in the BLM group compared with the control group, and HUMSCs-Ex decreased the IOD scores of Vimentin and increased the IOD scores of E-cadherin. Compared with the control group, a larger number of α-SMA^+^ myofibroblasts was found in the BLM group than in the control group. HUMSCs-Ex reduced the number of α-SMA^+^ myofibroblasts. The above changes in Vimentin, E-cadherin and α-SMA were similar at 1 week, 2 weeks and 3 weeks (Fig. 3).

### HUMSCs-Ex regulated the balance of M1/M2 macrophages in a mouse model of scleroderma.

**Fig. 4:**
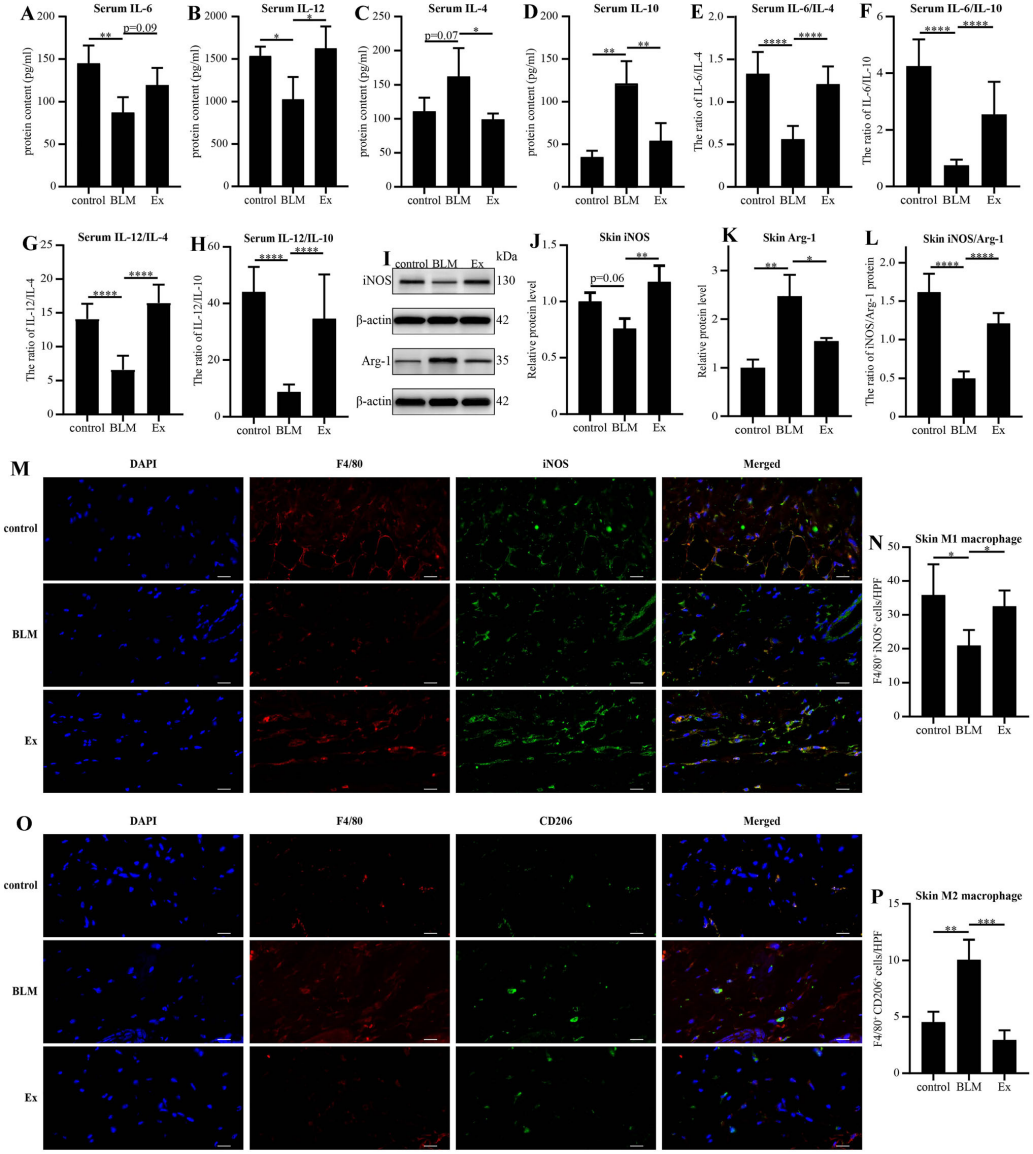
HUMSCs-Ex modulated the balance of M1/M2 macrophages. **A**–**D** The ELISA results of the inflammatory cytokines IL-6, IL-12, IL-4 and IL-10 in serum. **E**–**H** The ratio of IL-6/IL-4, IL-6/IL-10, IL-12/IL-4 and IL-12/IL-10 protein. **I**–**K** Western blot and quantification of iNOS and Arg-1 in skin. **L** The ratio of iNOS/Arg-1 protein. **M**–**P** Skin sections were stained with anti-F4/80 together with anti-iNOS or anti-CD206. Scale bars = 20 μm. The number of double-positive cells was counted under each high power field. Data are presented as the mean ± SD, *p < 0.05

To study the effect of HUMSCs-Ex on the balance of M1/M2 macrophages, skin and serum samples of mice killed in the first week were tested for related indicators. Compared with the control group, the protein levels of IL-6 and IL-12 secreted by M1 macrophages were downregulated in the BLM group. In contrast, the protein levels increased in the Ex group, but the changes in IL-6 were not statistically significant (*p* > 0.05 vs. the BLM group) (Fig. 4). The protein expression levels of IL-4 and IL-10 secreted by M2 macrophages were upregulated in the BLM group, but there was no statistically significant difference in IL-4 (*p* > 0.05 vs. the BLM group). HUMSCs-Ex reduced the protein expression levels of IL-4 and IL-10 (Fig. 4C, D). As shown in Fig. 4I, J, the result of Western blot results showed that the M1 macrophage polarization marker iNOS was upregulated and the M2 polarization macrophages marker Arg-1 was downregulated after HUMSCs-Ex interference in a mouse model of scleroderma. To reflect the status of M1/M2 macrophages more clearly, we took the ratios of their related markers. M1 macrophages were depolarized, M2 macrophages were polarized in the BLM group, and HUMSCs-Ex repaired the destroyed balance (Fig. E–H and L) (*p* < 0.05 the BLM group vs. the control group; *p* < 0.05 the BLM group vs. the Ex group). Compared with the control group, the number of F4/80^+^ iNOS^+^ positive cells (M1 macrophages) decreased in the BLM group. HUMSCs-Ex treatment increased the number of M1 macrophages (Fig. 4M–N). Moreover, the number of F4/80^+^ CD206^+^ positive cells (M2 macrophages) increased in the BLM group, and was attenuated by HUMSCs-Ex treatment (Fig. 4O–P).

## Discussion

At present, combination treatment with methotrexate and corticosteroids is a conventional treatment for scleroderma, but it is associated with severe side effects such as pancytopenia [15]. Although biologic agents are playing an increasingly important role in the treatment of scleroderma, these therapies cannot reverse the progression of the disease [16]. Therefore, there is an urgent need to seek a safe and effective treatment for scleroderma. In this study, we established a mouse model of scleroderma as the object of study. As a rare disease, scleroderma is characterized by clinical heterogeneity, which is not conducive to experimental research. The bleomycin-induced mouse model of scleroderma can reflect the features of human scleroderma well, with simple establishment and high reproducibility [17, 18].

Although some studies have been conducted on MSCs for scleroderma, there are few reports of their use in the clinic. One of the existing studies shows that three months after a patient with refractory scleroderma injected with MSCs, the patient's ulcerations decreased and the blood circulation of their hands improved [19]; another study followed five patients for 44 months after receiving MSCs, with skin improvement occurring in three patients, and no adverse events occurring in five patients [20]. However, those studies lack large multicenter randomized controlled trials. While we cannot be completely sure of the therapeutic efficacy of MSCs, these studies suggest the possibility of new therapeutic approaches.

Studies have shown that bone marrow mesenchymal stem cells or adipose mesenchymal stem cells [21] or their exosomes [22] achieve good therapeutic effects in the treatment of sclerodermatous mice, but there is no report on HUMSCs-Ex. Almost all cells can release exosomes, but umbilical cord mesenchymal stem cells are cost-effective, productive and acceptable to gain [23]. As a group of MSCs derived from neonatal umbilical cord, umbilical cord mesenchymal stem cells have a stronger ability to differentiate [24]. Therefore, we used HUMSCs-Ex as a tool to investigate the possibility of treating scleroderma.

We observed that HUMSCs-Ex alleviated bleomycin-induced skin fibrosis for at least 3 weeks. Additionally, we identified the key proteins α-SMA, Vimentin, and E-cadherin, and found that HUMSCs-Ex inhibited the transition from the endothelial form to the mesenchymal form. Takahashi [25] analyzed the skin lesions of 11 patients with scleroderma and found that α-SMA expression increased and E-cadherin expression decreased. Although HUMSCs-Ex has been described in some literature as having a similar ability to influence EMT [26, 27], this is the first time that HUMSCs-Ex have been discovered to regulate EMT in skin fibrosis. Organ fibrosis is the most important pathophysiological process of scleroderma, but vascular damage and immune disorders precede fibrosis [28]. Some scholars even believe that vascular injury can represent the pathogenesis of scleroderma, which is the basis of fibrosis [29]. A variety of cells, such as vascular endothelial cells and vascular smooth muscle cells, can be transformed into myofibroblasts through the EMT pathway, and the activated myofibroblasts can then synthesize collagen to result in ECM [30], which may express the interaction between proliferative vasculopathy and cutaneous fibrosis and their effect on the occurrence of scleroderma.

Activated macrophages can trigger a cascade of immune responses, and the loss of the balance of M1/M2 macrophages can lead to the occurrence of diseases. The most typical examples are M1 macrophages, which can cause acute inflammation and tissue damage, and M2 macrophages, which are involved in wound healing and induce fibrosis [31]. Skin CD204 and CD163, both phenotype markers of M2 macrophages, were significantly higher in scleroderma patients than in healthy subjects [32, 33]. The elevation of serum CD163 in scleroderma patients was closely related to disease severity [34]. Several drugs have been found to treat scleroderma mice by inhibiting M2 macrophage polarization, such as nintedanib [34], glycyrrhizin [35], tamibarotene [36], anti-CX3 CL1 mAb [37] and WKYMVM [38]. We found that HUMSCs-Ex elevated M1 macrophage markers (IL-6, IL-12p40, iNOS) and inhibited M2 macrophage markers (IL-4, IL-10, Arg-1 and CD206) in a mouse model of scleroderma, thus suggesting that HUMSCs-Ex might play an antifibrotic role by regulating the M1/M2 macrophage balance.

In conclusion, HUMSCs-Ex stimulated macrophage phenotypes from M2 to M1 and suppressed EMT, which might explain why HUMSCs-Ex played an antifibrotic role in scleroderma. For the first time, our study provided preliminary evidence for the clinical treatment of HUMSCs-Ex in scleroderma.

## Supplementary Information

Below is the link to the electronic supplementary material.

Supplementary file1 (RAR 1213 kb)
